# Nutrition-Oriented Reformulation of Extruded Cereals and Associated Environmental Footprint: A Case Study

**DOI:** 10.3390/foods9091260

**Published:** 2020-09-08

**Authors:** Namy Espinoza-Orias, Antonis Vlassopoulos, Gabriel Masset

**Affiliations:** 1 Société des Produits Nestlé S.A., Vers-Chez-les-Blanc, CP44, CH-1000 Lausanne 26, Switzerland; NamyDaniela.EspinozaOrias@rdls.nestle.com; 2 Vlassopoulos, Nutrition, Diet & Scientific Consulting, Ellispontou 35, 16232 Athens, Greece; avlassopoulos@aua.gr; 3 Cereals Partners Worldwide, Chemin du Viaduc 1, CH-1008 Prilly, Switzerland

**Keywords:** environmental impact, breakfast cereals, reformulation, life-cycle assessment

## Abstract

The global food system faces a dual challenge for the decades ahead: to (re)formulate foods capable to feed a growing population while reducing their environmental footprint. In this analysis, nutritional composition, recipe, and sourcing data were analyzed alongside five environmental indicators: climate change (CC), freshwater consumption scarcity (FWCS), abiotic resource depletion (ARD), land use impacts on biodiversity (LUIB), and impacts on ecosphere/ecosystems quality (IEEQ) to assess improvement after three reformulation cycles (2003, 2010, 2018) in three extruded breakfast cereals. A life cycle assessment (LCA) was performed using life cycle inventory (LCI) composed by both primary data from the manufacturer and secondary data from usual third-party LCI datasets. Reformulation led to improved nutritional quality for all three products. In terms of environmental impact, improvements were observed for the CC, ARD, and IEEQ indicators, with average reductions of 12%, 14%, and 2% between 2003 and 2018, respectively. Conversely, the FWCS and LUIB indicators were increased by 57% and 70%, respectively. For all indicators but ARD, ingredients contributed most to the environmental impact. This study highlights the need for further focus on the selection of less demanding ingredients and improvements in agricultural practices in order to achieve environmental and nutritional improvements.

## 1. Background

The global food system faces a dual challenge for the decades ahead: providing nutritious food to a growing population while reducing its environmental footprint [1,2,3]. Despite not being a new concept, the environmental sustainability of food is attracting renewed attention with the increased realization that sustainable food production will require extensive changes across the food system (e.g., agriculture, production, packaging, and consumption) [1], and that a healthy and sustainable diet, as proposed in the latest guidelines, is likely too expensive for 1.58 billion people globally [4].

Public health interventions in nutrition have traditionally been designed along the principles of two complementary approaches: the issuing of food-based guidelines and the theory of incremental changes [5]. The first encourages individuals to adopt healthier diets overall, i.e., changing their eating habits towards more plant-based products, and products with lower levels of saturated fats, sugar and sodium [1,6,7]. The second focuses on the improvement of existing products and production methods, mainly through product reformulation, and it is aimed as a short-to-medium term action that respects consumer expectations, avoids the inflation of a diet’s cost, and as such, delivers improvements even among the lowest socioeconomic classes [8,9].

While there is extensive evidence on the association between healthier and more environmentally friendly dietary choices overall [1,6], recent evidence suggests that dietary shifts alone, i.e., the first approach, may not suffice to fit the global food system within the ‘planetary boundaries’ [10]. Improvements of the production methods are needed to further reduce the environmental footprint of the food system. Studies looking at specific food products, their process and/or nutritional properties, have rarely combined environmental and nutritional dimensions together. Indeed, studies tend to focus on improvement of the food production per se, highlighting alternative methods (regenerative agriculture, crop rotation, use of crop cover, use of organic manure and less synthetic fertilizers, no tillage) to reduce the environmental footprint of products [11,12,13,14]; or focusing on the evolution of the nutritional profile of specific food products, or group of food products, and their potential impact of nutritional intakes and health outcomes [8,15,16,17]. The question of whether such reformulations were compatible with a reduction of the environmental footprint of the food system has been rarely addressed, but the results are so far promising [18,19,20,21]. However, the study of food sustainability presents challenges and would require the wider scientific community to become familiar with unit efficiency in food production and life-cycle assessments, as well as understanding that environmental footprint analysis goes beyond the commonly used greenhouse gas emissions to include multiple indicators (e.g., eutrophication, acidification, ozone depletion, land use, water scarcity, ecotoxicity, photochemical ozone formation, respiratory organics/inorganics, abiotic resource depletion, ionizing radiation) [22,23,24].

This paper aims to expand existing literature on food sustainability by presenting three cases of food reformulation in extruded breakfast cereals—carried out with the purpose of improving their nutritional value—and its impact on the products’ environmental impacts. The latter were assessed through a complete life cycle assessment, including multiple sustainability indicators and presenting 15-years′ worth (2003–2010–2018) of primary data from the three most produced recipes by Cereal Partners France. The aim of this analysis was to assess whether product reformulation could be an opportunity to improve a product’s environmental footprint.

## 2. Methods

### 2.1. Cases Selection

Three recipes of extruded breakfast cereals with the highest sales volume (per 2018 sales) manufactured by Cereal Partners France were selected. Cereal Partners France (CPF), a division of Cereal Partners Worldwide (CPW), manufactures breakfast cereals for domestic consumption at two facilities in France. Primary data was collected from CPF for three individual years over a 15-year period, namely: 2003, 2010, and 2018. Even though product reformulation cycles may be shorter, these three years represent key changes in nutritional profile changes of the products, in particular the inclusion of whole grains in the recipes, the reduction of sugar content, and the removal of palm oil. Data from the 2003 recipes serve as the baseline while data from the next two major recipe changes in 2010 and 2018 serve as examples of the intermediate and current recipes. For all products, only the main recipe was considered excluding any alternative products with different flavors or formats. The products analyzed were Chocapic^®^ 430 g, Nesquik^®^ 450 g, and Lion^®^ 480 g, produced in the Rumilly and Itancourt factories of Cereal Partners France (hereafter mentioned as Cereals 1–3, respectively. Detailed description of the product and packaging, recipes, and nutritional compositions are available in Supplementary Materials Figure S1A–C, Tables S1–S3, and Table S8).

### 2.2. Life Cycle Assessment Methodology

**Figure 1:**
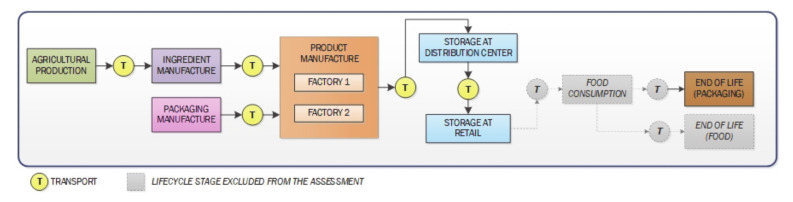
Scope of the study and boundaries of the breakfast cereals product system.

The functional unit chosen for the study is the “consumption of one serving size of breakfast cereal (30 g)”. Figure 1 describes the “cradle to grave” boundaries defined for the system under assessment. The system includes the agricultural production of ingredients, production of packaging materials, manufacture of breakfast cereals, distribution, retail, end of life of packaging materials, and all transportation in between life cycle stages. Two main life cycle stages are excluded from the assessment, namely consumption of cereals with milk or alternative products (plant-based beverages such as “soy milk”, “almond milk”, “oat milk”, or “pea milk”) and end of life management of post-consumer breakfast cereal waste. The reason to exclude the consumption stage lies in the fact that the nutrients provided by the combination of a serving size of breakfast cereal and a beverage (milk or a plant-based beverage) can differ greatly, rendering the potential scenarios for assessment not functionally comparable (understood as delivering the same nutrient-provision function). Similarly, the amount of post-consumer food waste depends on consumer behavior. Transportation of the product from retail store to home and of waste from home to end of life treatment facility are excluded because these are not significant contributors to the overall life cycle of the product. All transportation steps between life cycle stages until shop gate were accounted for in the model. Transportation of the product from shop to home and of waste packaging from home to end of life management location were excluded from the model because their contribution to the overall results fall below a materiality threshold of 1% (ranging between 0.01% and 0.5% for all impacts evaluated).

Life cycle inventory included data on recipe formulation, packaging, sourcing of materials, manufacture, distribution, and retail as provided by the manufacturer. Secondary data coming from regional statistics was used to model end of life management of packaging materials in France. Life cycle inventory datasets are sourced from the following databases: ecoinvent v.3.4, allocation based on recycled content (2017) and World Food LCA Database (WFLDB) v.3.3 (2017) (Quantis SARL, Lausanne, Switzerland). The LCA was carried out using the life cycle assessment software SimaPro v.9.0 (PRé Sustainability B.V., Amersfoort, The Netherlands).

### 2.3. Nutritional Data and Raw Material

The exact recipes and ingredient lists for the production of 100 g of finished product was provided by the manufacturer (Cereal Partners France) and closely matched the labeled ingredient list with the exception of humidity lost during production due to drying of the product (Supplementary Materials Tables S1–S3). Region of origin was provided for all raw materials and it was assumed that agricultural production of the ingredients followed conventional agricultural practices. Cereals and sugar are sourced largely from France; palm oil and palm kernel oil are sourced from South East Asia; soybean oil and soy lecithin are sourced from South America; and the remaining ingredients are sourced from various European locations (Supplementary Materials Table S4). For the purpose of this analysis, it was assumed that suppliers remained unchanged over the period of the study. The nutritional composition of the products was based on the nutritional declaration as labelled. The Nutri-Score algorithm was used to assess the nutritional quality of the products at each time point [25].

### 2.4. Environmental Impact Assessment

Five environmental impact indicators were evaluated in the study, chosen because they provide a sufficient coverage of relevant environmental issues associated with packaged food products:

- Climate change (kg CO_2_-eq, eq = equivalent here and below) (IPCC 2013, 100 a) [26]. Measures the greenhouse gases emitted as part of agricultural processes (application of fertilizers, livestock enteric fermentation), land use change, combustion of fuels and waste, anaerobic decay of bio-based materials, and industrial processes. Biogenic CO_2_ is assigned a characterization factor of 0. This is a midpoint indicator.
- Freshwater consumption scarcity (m^3^-eq) [27]. Measures the availability of water for use downstream, weighted by local water scarcity factors. This is a midpoint indicator.
- Abiotic resource depletion (kg Sb-eq) (CML 2001 method, v. 2.05; CML, Center of Environmental Science, Leiden University, The Netherlands) [28]. Measures the potential for depletion of non-renewable resources in relation to a reference substance (antimony, Sb). This is a midpoint indicator.
- Land use impacts on biodiversity (PDF × m^2^ × year; PDF = Potentially Disappeared Fraction). Measures the impact of different land uses (arable, permanent crops, pasture, forestry, fallow land, industrial, traffic area, urban) on the biodiversity present in that area over a given period (IMPACT World+/Land use method, v.0.05) [29]. This is an endpoint indicator.
- Impacts on ecosphere/ecosystems quality (PDF × m^2^ × year) (IMPACT 2002+ method v. Q2.27) [30,31]. This is a compound indicator comprising three indicators: eutrophication, ecotoxicity, and acidification. Eutrophication is caused by over-enrichment of aquatic environments with nutrients, typically N and P. Acidification is caused by the deposition of S and N in the soil, thus, affecting its buffer capacity, causing imbalances in the soil composition and affecting its pH. Ecotoxicity refers to the effect on the biota caused by toxic substances emitted into the environment (air, soil, water bodies), such as pesticides, fertilizers, heavy metals, volatile compounds. This is an endpoint indicator.

An increase in either of these indicators signifies a larger impact in the respective environmental components and, hence, a larger burden to the environment.

### 2.5. Inclusion of Packaging, Manufacture, Distribution and Retail, and of Life and Transportation in the Life Cycle Inventory

Primary data on packaging was used for the LCA calculated as g of material used per cereal box (Supplementary Materials Table S5). In this case, primary packaging corresponds to the “bag in box” format, containing between 430 and 480 g of breakfast cereal. This packaging format is required to protect the brittle nature of the product. These boxes are then packed into secondary packaging made of corrugated board (between 12 and 22 boxes per case). The cases (between 16 and 24) are loaded in layers onto pallets and wrapped with stretch film (tertiary packaging). Plastic materials used include, high-density polyethylene (HDPE) and linear low-density polyethylene (LLDPE) sourced from the UK; folding board boxes (FBB) and corrugated board cases sourced from the UK and France. Virgin materials are used in the packaging system (plastic and fiber based). Other packaging, used for some raw ingredients and packaging materials, is excluded from the assessment. Cereals and flours are delivered in bulk at factory gate. No changes in distribution packaging occurred during the reformulation process.

Data corresponding to the overall annual operation at two factories in France for the three years (2003, 2010, 2018) were used to estimate the impact of manufacture on the environmental footprint of the products. The manufacturing process data were calculated per kg of produced products and as such annual production tonnage and investment on projects aiming to improve energy and water efficiency were taken into account (Supplementary Materials Table S6). Waste is also generated during manufacturing, and detailed records exists for years 2010 and 2018, indicating the various waste streams and the respective end of life management options taken. However, after calculating the ratio of mass of waste to mass of product manufactured, it was decided not to include these datasets in the assessment due to them being non-material (ranging in the order of 10^−5^ to 10^−4^ kg per kg of final product).

For distribution and retail, the LCA model operated under the assumption that breakfast cereals are ambient, shelf-stable products, with a typical shelf life of 9 months. Once manufactured, products leave the factories and are taken to regional distribution centers, where they are stored for 6 days. Then, they are taken to retail stores where the typical turn around period is 5 days. All transportation steps between life cycle stages were accounted for in the model.

The end of life assessment included only packaging material. Primary packing (the packaging used for the cereal boxes themselves) includes four waste channels: municipal solid waste landfilling and incineration, plastic packaging recycling and cardboard recycling. Data from national and European Union statistics were used to estimate how municipal solid waste and specific packaging materials were treated in France over the years of the assessment (2003, 2010, and 2018; full details in Supplementary Materials Table S7) [32,33,34]. In brief, it was assumed that in the years 2003 and 2010, plastic bags were not recycled and were instead treated as municipal solid waste, and composting was not included as a disposal option at any time point. For secondary packaging (packaging used for transport and storage) it was assumed that secondary cardboard was recycled at a 100% rate while secondary plastic was treated under the same conditions as primary plastic.

Given the major contribution of land use change to climate change, a sensitivity assessment was carried out to evaluate the effect of improved agricultural practices on this indicator. Conventional agricultural practices take into account statistical records of actual deforestation in the cultivation of three key commodities whose processed ingredients are part of the recipes of the breakfast cereals assessed: soybean oil, cocoa powder, and palm oil. On the other hand, improved agricultural practices consider that no land use change would have taken place in the cultivation of said commodities. This was possible by using life cycle inventories from the World Food LCA Database (v.3.3), which incorporate country or commodity specific factors for land use change.

### 2.6. Statistical Analysis

The aim of the study was to identify potential gains in multiple environmental indicators using real-life in a modeling scenario in three different products all reformulated in the same manner. Given that the sample selected for the analysis is a fixed sample of products (case study) and not meant to represent all products under the same category, only descriptive statistics were used to identify changes in key indicators. In contrary to direct sampling, modeling scenarios, such as the one employed in this analysis, do not provide data with true variability, and all results are to be used comparatively.

## 3. Results

### 3.1. Raw Materials and Nutritional Composition

**Figure 2:**
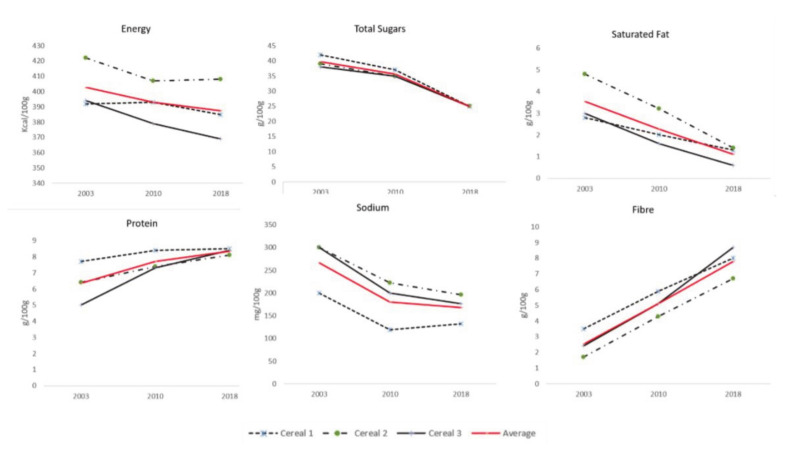
Changes in the nutritional composition of the products studied over the 2003–2018 period.

In the previous 15 years, the recipes of the products studied changed as a result of product reformulation (details in Supplementary Materials Tables S1–S3). In terms of ingredient choices and recipes, the major changes include the introduction of wholegrains, since 2010, as a substitute of the previously used refined grains. Between 2003 and 2018, the use of refined grains was reduced by 45% in average and wholegrains were introduced in amounts that covered the reduction in refined grains by 1.45 times. Overall, there was an overall 60% reduction in the use of fats and oils. Alongside the reduction, there was a complete removal of palm oil by 2018 and a substitution by sunflower oil as the main oil used. Sugars from all sources were reduced by 10% in the 15 years studied, and the use of cocoa powder or cocoa mass remained unchanged. The main cereals used include wheat and corn, with rice removed from the ingredients since 2010. As shown in Figure 2 and Supplementary Materials Table S8, the main changes in the nutritional composition were a more than two-fold increase in the mean fiber content, followed by a nearly 70% reduction in mean saturated fat and a 37% reduction in mean sugar and sodium content. Protein was also increased by 31% on average while the energy content remained largely unchanged (<6% reduction for all).

**Figure 3:**
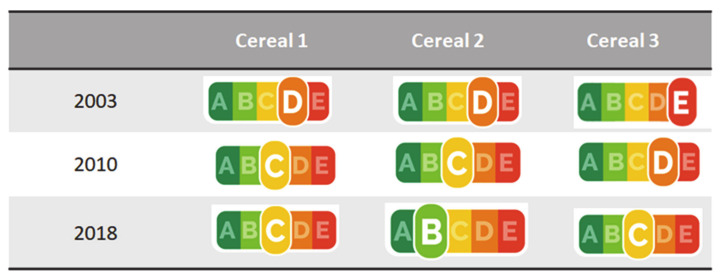
Changes in the Nutri-Score rating of the products studied over the 2003–2018 period. A represents higher nutritional quality, and E lower nutritional quality.

The changes in the nutritional composition were reflected in an improvement of the Nutri-Score rating of all three products (Figure 3).

### 3.2. Environmental Footprint Assessment

**Figure 4:**
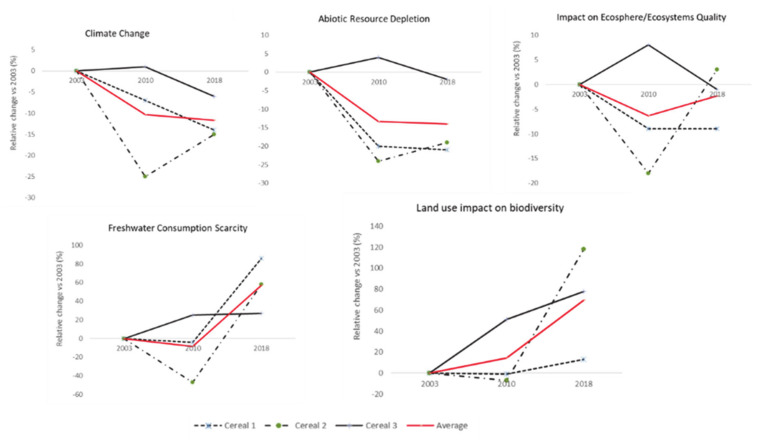
Changes in environmental indicators of the products studied over the 2003–2018 period.

In terms of changes in the environmental footprint of the cereal products studied, the analysis indicates a mixed picture (Figure 4). On average, the three products showed improvements for the climate change (mainly greenhouse gas emissions) and abiotic resource depletion indicators, with an average impact reduction in the range of 12% and 14%, respectively. On the other hand, smaller improvements were seen for impact on the ecosystem with a 2% reduction of the indicator and only one of the three products showing consistent improvements through the study period. Conversely, all three products showed higher needs for freshwater usage and a greater impact on the biodiversity due to land use with average increases of 57% and 70%, respectively.

For all scenarios assessed, changes in freshwater usages were attributed to irrigation of crops in different countries (83% to 93% of the results of this indicator), and other uses account for the remaining 7% to 16% of impact (data not shown). Agriculture and especially the use of land for agricultural purposes explains between 85% and 97% of all impact in biodiversity. In particular, land use impacts on biodiversity are driven by occupation of land for the cultivation of annual crops such as cereals (52% to 94%), and perennial crops such as cocoa beans (3% to 32%).

A reduced use of resources such as solid fossil fuels (ranging between 25% and 30%), natural gas (ranging between 40% and 48%), or crude oil (ranging between 26% and 35%) explain changes in the abiotic resources′ depletion. Similarly, there are four main drivers behind 90% of the impact on ecosystem quality in all scenarios. The largest contributor is ammonia (ranging between 61% and 67%) due to its characterization in aquatic and terrestrial ecotoxicity and aquatic and terrestrial acidification. The second largest contributor are nitrous oxides, ranging between 14% and 16% and characterized in terrestrial and aquatic acidification. The third largest contributor is phosphate, ranging between 9% and 10% and characterized only in aquatic eutrophication. Finally, sulfur dioxide contributes between 2% and 3% and is characterized in aquatic and terrestrial acidification.

On the contrary, the impact on climate change could be equally attributed to greenhouse gases (CO_2_, CO, and CH_4_) arising from land transformation and greenhouse gases arising from industrial processes (N_2_O and fossil CO_2_, CO, and CH_4_) for two out of three cases (46–55% land transformation to 43%–50% industrial processes), but not for the third case in which greenhouse gases from industrial processes explain 62–65% of the climate change impact (data not shown).

**Figure 5:**
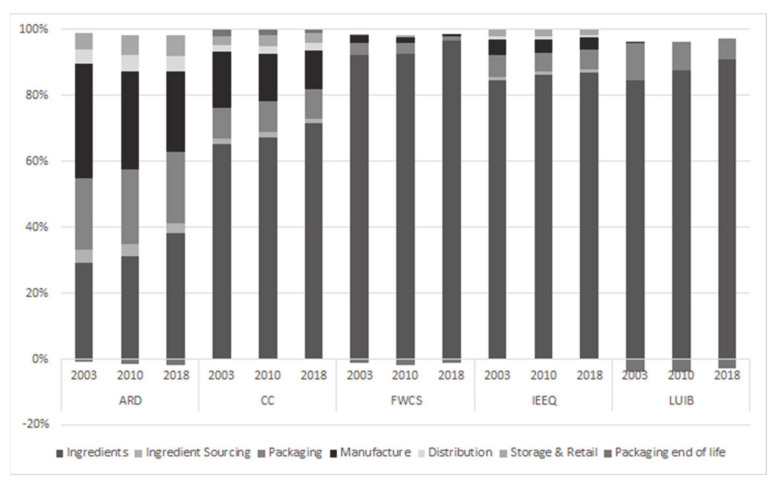
Contribution of each life cycle assessment (LCA) stage on the overall environmental indicator (data for the average product, *n* = 3); CC: climate change; ARD: abiotic resource depletion; LUIB: land use impact on biodiversity; IEEQ: impact on ecosphere/ecosystems quality; FWCS: freshwater consumption scarcity.

For all indicators, except abiotic resource depletion, the choice of ingredients was the main contributor towards the environmental footprint of the average product explaining 64%-98% of all variability per indicator (Figure 5 and Supplementary Materials Tables S9–S11). For abiotic resource depletion, both the choice of ingredient and manufacture had equal contribution of 25%–40%, followed by packaging that explained ≈20% of the impact. Packaging was also responsible for 6.6–12% of the impact on land use on biodiversity and both packaging and manufacture contributed ~10% of the total climate change impact. Interestingly, although the total impact on climate is reduced for all products from 2003 to 2018, the importance of ingredient choice as the main contributor towards carbon footprint is increasing from 64,7% in 2003 to 71% in 2018. This could be linked to the reduced contribution of manufacturing towards the products’ carbon footprint from 17% to 11.7% from 2003 to 2018. In the case of abiotic resource depletion (ARD), land use impacts on biodiversity (LUIB), and freshwater consumption scarcity (FWCS), negative numbers are explained by the fact that the packaging materials are recycled or incinerated with energy recovery, thus reducing the need to use virgin materials or use fossil fuels to generate district heat. Negative numbers imply a “credit to the system”. Recycling cardboard can be attributed as the contributor to this (forestland spared and water used in pulp processing spared).

Given that ingredients were shown to be the major contributor towards the environmental footprint for most indicators, a secondary analysis was carried out to measure the changes in all environmental indicators attributable only to ingredient selection (Table 1). Studying the changes in ingredients alone, the evolution of the environmental indicators was in the same direction as the complete LCA analysis—albeit to a smaller degree—with the exception of abiotic resource depletion and impact on the ecosphere quality. For those two indicators that are shown to have improved in the complete LCA analysis, it would appear that changes in steps further down the product life cycles have larger effects that mandate the net impact (likely manufacture and packaging). The same can be said for the climate change indicator whose 15-year improvement is 4-fold larger in the complete LCA as compared to when analyzing ingredients alone. 

As ingredients are the main contributors to the carbon footprint, and given the above stated results, a sensitivity analysis was carried out to measure the impact of changing agricultural practices on the carbon footprint of the products. As shown in Table 2, switching from ingredients that have been produced with conventional practices to those following practices that do not promote deforestation can reduce the estimated CO_2_ emissions of a single serving by 21% to 55%, which highlight the significance of sourcing commodities from supply chains where increased traceability can demonstrate adherence to no deforestation commitments. 

## 4. Discussion

This study aimed to use real-life examples to estimate the potential to improve extruded breakfast cereal environmental impact via nutrition-focused reformulation.

The recipe changes that occurred during the 15-year reformulation cycle, i.e., switching from palm oil to sunflower oil and from purely refined cereals to a mix of wholegrain and refined cereals alongside a general reformulation to reduce sugar and fat content, led to substantial improvements in the products’ nutritional value as measured by Nutri-Score. On the level of individual nutrients, the recipe changes were linked to reductions in energy, total sugars, saturated fats, and sodium content with parallel improvements in their fiber and protein content.

The reformulation cycle, together with the changes that occurred in the other stages of the supply chain, was associated with improvements of three environmental impact indicators out of the five assessed in this study (climate change, abiotic resource depletion and impact on ecosphere quality); with increases observed for the land use impact on biodiversity and freshwater consumption scarcity. 

Ingredients (i.e., the agricultural stage) contributed to more than 60% of the environmental impact for all indicators but abiotic resource depletion, at all phases of the reformulation cycle. In this specific study, the removal of rice, potentially one of the crops with the highest carbon footprints [35], and the switch from palm oil to sunflower oil could explain the changes in carbon footprint seen both at the ingredients level and for the full LCA [36,37]. However, despite the high irrigation needs of rice [38], its removal was not linked to improvements in freshwater scarcity. Similarly, the removal of palm oil was not associated with improvements in markers on biodiversity protection. A potential explanation for those changes could be linked to the hypothesis made in this analysis that all ingredients were produced following conventional practices. As shown in the sensitivity analysis, changes in the agricultural practices towards a no deforestation policy could have a high impact on the greenhouse gas emissions of these products (up to 55% reductions); and those same agricultural practices would promote greater biodiversity protection. The present results thereby strengthen the rationale for increasing the effort on improving farming practices to reduce the environmental impact of the food system [39,40]. The food industry has a key role to play in promoting those beneficial agricultural practices via its choice of suppliers and the promotion of agricultural transformation. While the choice of suppliers based on their environmental footprint may have attracted little attention so far, data indicate that even within the same country, the region of production for a raw material is linked with great differences in its carbon and water footprint, under the same agricultural practices [41]. In reality though, the implementation of this environmental management strategy requires large resources in terms of establishing monitoring and auditing protocols, increasing the frequency of site visits, creating and strengthening partnerships with external verification organizations and local authorities, and investing in new technological solutions combining satellite remote sensing and big data analytics [42].

The contribution of ingredients to the environmental impact of products increased throughout the reformulation cycle. This was due to the reduction—in absolute terms—of the environmental impact of the other stages of the life cycle, in particular manufacturing, packaging, and end-of-life. These improvements, which partly mitigated some negative effects of ingredient changes, highlight the increased rates of recycling that occurred in France in the 15 years analyzed, and the continuous progress that has been made by food manufacturers in terms of efficiency of manufacturing and packaging technology.

This analysis is not without caveats. Firstly, the product category studied is unique in terms of its environmental impact as the stage of transport, retail and food waste by the consumers are fairly small contributors in the environmental footprint given that the products are dried and shelf stable. The same analysis could yield substantially different results if carried out in products that require a cold-supply chain [43]. Even among the cereal-based products, breakfast cereals have different LCAs than products like bread in which food waste and storage would play a larger role in the overall carbon footprint despite ingredients remaining the main contributor [44]. Moreover, the choice of databases to retrieve the environmental data and the assumptions made for the suppliers and manufacturing methods of the historical recipes indicate that extrapolation of these results to other food categories should be made with caution [45,46,47]. The lack of regularly updated lice cycle inventory databases, which would include good quality historical and statistical data and the reliance on the publicly available datasets, hinders the capacity to reflect on actual changes in the agricultural practices [48]. On the other hand, this study is potentially the only one to present data on a full LCA analysis using a combination of publicly available and real-life data in all stages of the model and utilizing access to the manufacturers own databases to increase accuracy. In terms of carbon footprint, the current analysis estimated that greenhouse gas emissions of the analyzed products fall in the range of 67–100 g CO_2_-eq for every 30 g portion after reformulation. Previous studies that followed a similar methodology and carried out a full LCA analysis (bar end of life analysis) indicate that greenhouse gas emissions for cereal products fall in the range of 80–117 g CO_2_-eq, with exception of two studies that indicated much lower emissions 21–30 g CO_2_-eq, potentially due to the studies’ limited scope (studying specific aspects of the cereal product production or use of different life cycle inventory (LCI) databases) [11,49,50,51,52]. As with all LCA analyses, the results of this study should be treated with caution, as they are relevant to the specific products and the regions they are produced. The aim of such analyses is to identify hotspots across the life cycle of a product and provide an estimation of the relative impact on various environmental indicators. As such, these analyses provide with valuable insights in the options to improve a product’s environmental impact. Along those lines, our findings are in agreement with previous studies that identified similar hotspots and changes in the same range for similar indicators [11,49,50,51,52].

Overall, the results add to an ongoing discussion among academia, food industry, and the consumers on how food can become more sustainable and more nutritious in order to address future challenges. The results highlight that the supply chain as a whole and the raw materials in particular are key areas for future work. This observation also holds true for whole diets [53]: the Mediterranean diet, with its short supply chains, high utilization of low environmental impact agriculture, and respect for seasonality being a prime example of the need for a systems approach on food sustainability [54]. Consumers are increasingly concerned over the provenance and environmental performance of the food they consume, but they often seem to focus on one aspect of a product’s environmental impact like its packaging [55] or the so-called food mile, a metric of the greenhouse gas emissions related to the transportation of a food from production to the kitchen cupboard. Often environmental sustainability is intertwined with the notion that a food is necessarily better for health, or that it includes less unfamiliar ingredients, and it is produced in a more ‘natural’ manner [56,57,58,59]. The increased awareness of consumers around sustainability issues and the potential it has to impact consumer choices will require new research that expands beyond the simple greenhouse gas emissions metrics and the identification of new ways to communicate the complexity of an environmental impact LCA analysis to a non-expert audience [21].

Today, multiple environmental impact indicators exist, each focusing on a certain aspect of the environmental impact equation. As previously shown in the nutrition world, in order to successfully drive consumer behavior and engage multiple stakeholders, the creation of composite markers that weigh the different aspects and serve as a simple to use metric to compare one food to another will be needed [60,61,62]. The science of nutrient profiling was developed in nutrition for this purpose [63] and a similar discipline might soon be needed in the environmental sciences. Although primary reports exist on the conception and usefulness of such indicators [64], the creation of a unique metric that combines all aspects of a multi-indicator assessment will require a series of subjective choices which will eventually be challenged by the scientific community. The European Union Product Environmental Footprint methodology has attempted the creation of a framework to study simultaneously 15 environmental indicators, created a list of normalization factors and issued a series of pilot studies aiming in reducing the complexity of multi-indicator assessment towards an easier assessment and communication of the impact of products on the environment [65,66].

## 5. Conclusions

This study highlights that nutritional and environmental improvements in dry breakfast cereals are not always positively correlated. While ingredients contributed the most to the environmental impact of the analyzed products, the food industry has managed to reduce some of the environmental impact of such products mainly through improvements in post-agricultural life cycle stages. The results highlight the need for reformulation strategies that look beyond the factory gates and engage the agricultural sector in a way that promotes more sustainable production of raw material, improvements in its transportation, and even reduction of food losses in the field.

## Figures and Tables

**Table 1:** Changes in the five environmental impact indicators in the 2003–2018 period for the product average- analysis of ingredients alone versus the complete LCA.

Recipe Year	Ingredients Only					Complete LCA				
	ARD	CC	FWCS	IEEQ	LUIB	ARD	CC	FWCS	IEEQ	LUIB
2003	0%	0%	0%	0%	0%	0%	0%	0%	0%	0%
2010	−7%	−7%	−8%	−4%	18%	−13%	−10.3%	−8.7%	−6.3%	14.3%
2018	12%	−3%	62%	1%	78%	−14%	−11.7%	57%	−2.3%	69.7%

CC: climate change; ARD: abiotic resource depletion; LUIB: land use impact on biodiversity; IEEQ: impact on ecosphere/ecosystems quality; FWCS: freshwater consumption scarcity; LCA: life cycle assessment

**Table 2:** Effect of introduction of no deforestation practices over climate change impact results (kg CO_2_-eq/30 g serving size).

Product	Year	Conventional Practices	No Deforestation Practices	Potential Change
Cereal 1	2003	9.4×10^2^	5.0×10^2^	−47%
	2010	9.6×10^2^	5.4×10^2^	−44%
	2018	8.9×10^2^	5.2×10^2^	−41%
Cereal 2	2003	1.2×10	5.9×10^2^	−49%
	2010	1.1×10	4.8×10^2^	−55%
	2018	1.0×10	5.1×10^2^	−49%
Cereal 3	2003	9.0×10^2^	6.5×10^2^	−27%
	2010	6.7×10^2^	4.6×10^2^	−32%
	2018	7.6×10^2^	6.0×10^2^	−21%

## Supplementary Materials

The following are available online at https://www.mdpi.com/2304-8158/9/9/1260/s1, Table S1. Ingredients used in Cereal 1 breakfast cereal (period 2003–2018). (g ingredient needed/100 g finished product)—cut-off 0.15%; Table S2. Ingredients used in Cereal 2 breakfast cereal (period 2003–2018). (g ingredient needed/100 g finished product)—cut-off 0.15%; Table S3. Ingredients used in Cereal 3 breakfast cereal (period 2003–2018); (g ingredient needed/100 g finished product)—cut-off 0.15%; Table S4. Sourcing of materials; Table S5. Packaging system composition (g material/breakfast cereal box); Table S6. Manufacturing processes data (per kg of product); Table S7. End of life options for packaging materials in France; Table S8. Nutritional content of all breakfast cereal recipes; Table S9. Relative contribution of life cycle stages to overall impacts for Cereal 1; Table S10. Relative contribution of life cycle stages to overall impacts for Cereal 2; Table S11. Relative contribution of life cycle stages to overall impacts for Cereal 3; Figure S1A–C: Detailed presentation of products used in the analysis as indicated in the packaging (2018 format), Figure S2. Environmental impacts of one serving size (30 g) of three breakfast cereal products manufactured in France over a 15-year period (2003–2018).
