# Supplementary material for: Nutrition-Oriented Reformulation of Extruded Cereals and Associated Environmental Footprint: A Case Study
Source: Foods. 2020 Sep 8;9(9):1260. doi: 10.3390/foods9091260 (PMC7555475; doi:10.3390/foods9091260)
Supplement: Supplementary file 1 [file foods-09-01260-s001.pdf]

(A)

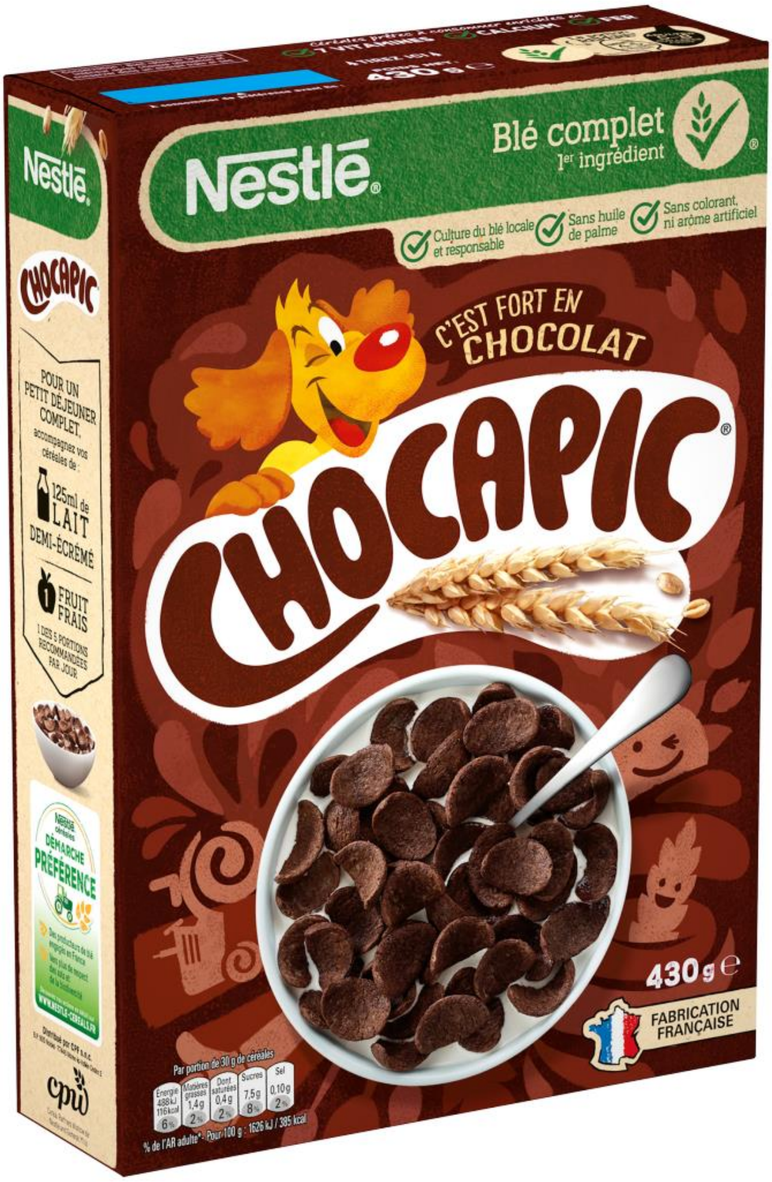
Nestlé
Blé complet
1er ingrédient
Culture du blé locale et responsable
Sans huile de palme
Sans colorant, ni arôme artificiel
C'EST FORT EN CHOCOLAT
CHOCAPIC
430 g ℮
FABRICATION FRANÇAISE
POUR UN PETIT DÉJEUNER COMPLET, accompagnez vos céréales de :
125ml de LAIT DEMI-ÉCRÉMÉ
1 FRUIT FRAIS
DÉMARCHE PRÉFÉRENCE

(B)

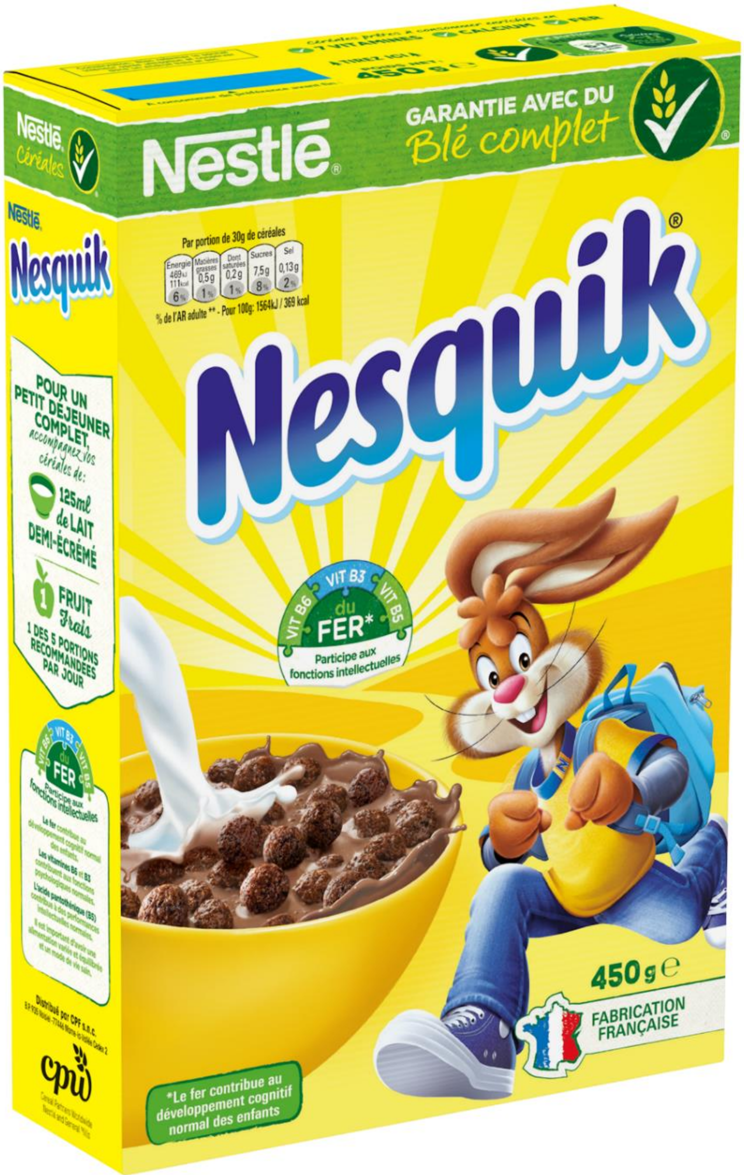
Nestlé
GARANTIE AVEC DU
Blé complet
Nesquik
Par portion de 30g de céréales
Nesquik
POUR UN PETIT DÉJEUNER COMPLET, accompagnez vos céréales de:
125ml de LAIT DEMI-ÉCRÉMÉ
1 FRUIT frais
1 DES 5 PORTIONS RECOMMANDÉES PAR JOUR
VIT B6
VIT B3
VIT B5
du FER*
Participe aux fonctions intellectuelles
450g ℮
FABRICATION FRANÇAISE
*Le fer contribue au développement cognitif normal des enfants
cpw

(**C**)

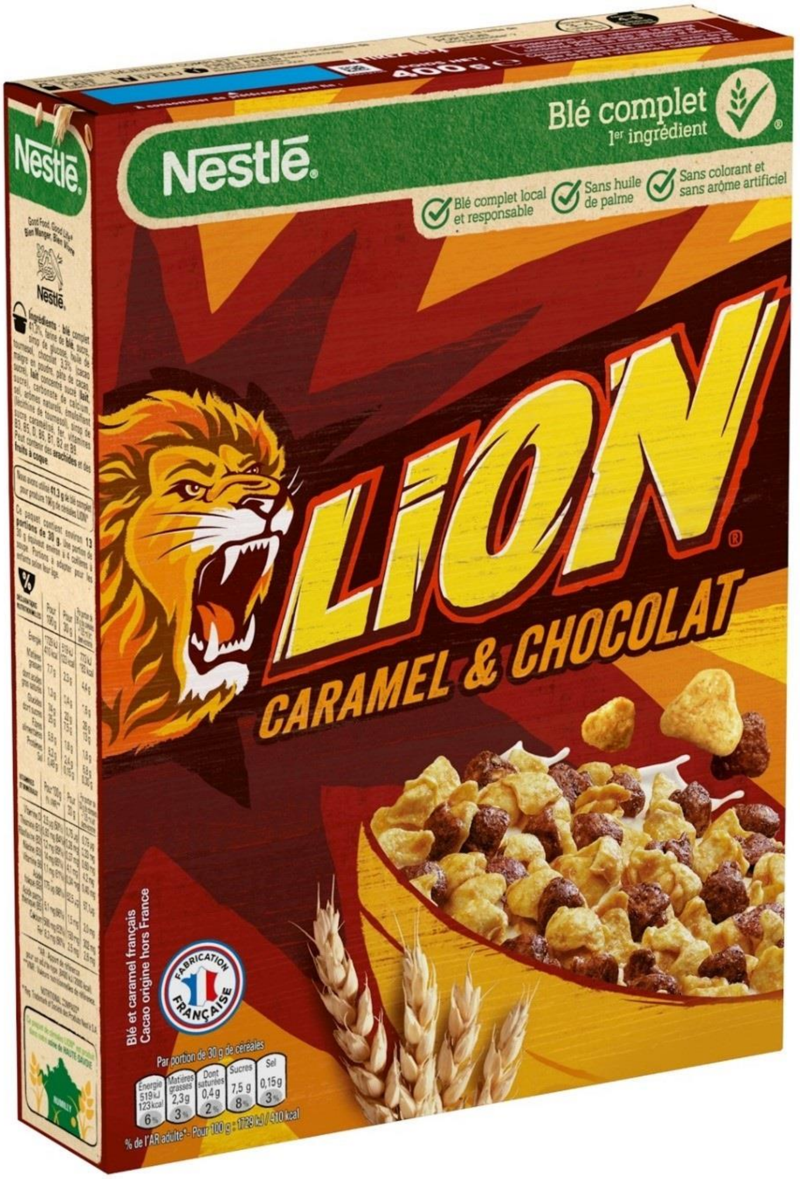

Figure S1. Detailed presentation of products used in the analysis as indicated in the packaging (2018 format). Cereal 1—Chocapic 430g (**A**); Cereal 2—Nesquik 450 g (**B**); Cereal 3—Lion 480 g (**C**).

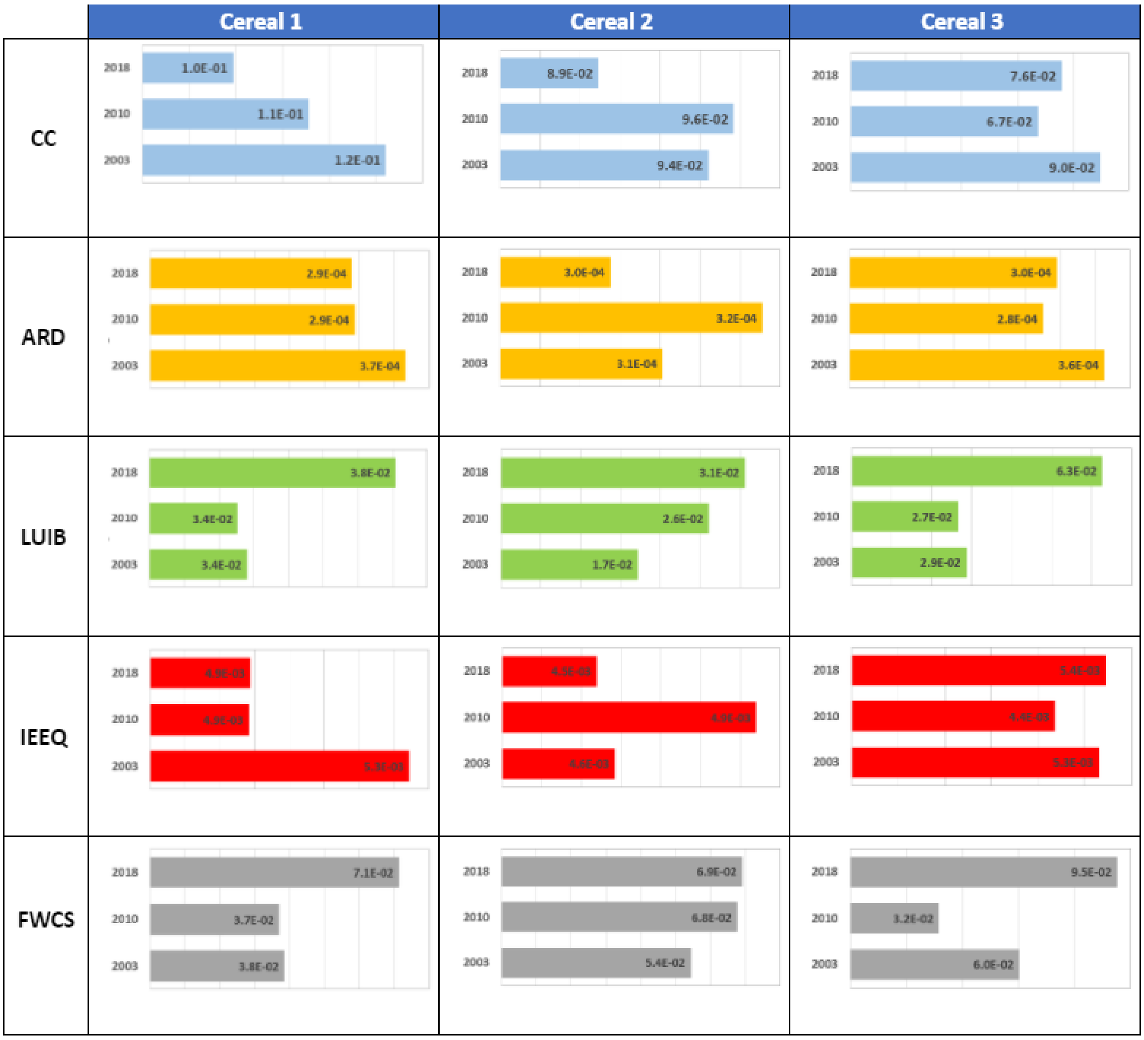

Figure S2. Environmental impacts of one serving size (30 g) of three breakfast cereal products manufactured in France over a 15 year period (2003–2018). CC: Climate change; ARD: Abiotic resource depletion; LUIB: Land use impact on biodiversity; IEEQ: Impact on ecosphere/ecosystems quality; FWCS: Freshwater consumption scarcity.

Table S1. Ingredients used in Chocapic (Cereal 1) breakfast cereal (period 2003–2018). (g ingredient needed / 100g finished product)—cut-off 0.15%.

Ingredients	2003 Recipe	2010 Recipe	2018 Recipe
Refined wheat flour	52.0	24.9	16.8
Whole wheat flour		31.9	33.5
Corn semolina			15.1
Sugar	38.0	32.6	21.8
Dextrose	4.01	3.62	
Glucose syrup			7.38
Cocoa powder	7.72	8.57	7.33
Palm oil	2.0	1.64	
Sunflower oil			1.65
Calcium carbonate	0.66	0.70	1.15
Salt	0.38	0.29	0.25
Soya lecithin	0.40	0.44	
Sunflower lecithin			0.43
Malt extract	0.18	2.13	

Table S2. Ingredients used in Nesquik (Cereal 2) breakfast cereal (period 2003–2018). (g ingredient needed / 100g finished product)—cut-off 0.15%.

Ingredients	2003 Recipe	2010 Recipe	2018 Recipe
Rice flour	34.3	6.17	
Corn semolina	18.1	23.7	17.0
Wheat starch	5.0		
Whole wheat flour		31.8	51.4
Sugar	35.3	32.8	23.1
Dextrose	2.85	1.98	
Glucose syrup			11.3
Cocoa powder	5.63	6.02	5.68
Palm oil	2.85	1.66	
Calcium carbonate	0.67	0.70	1.12
Salt	0.70	0.47	0.43

Table S3. Ingredients used in Lion (Cereal 3) breakfast cereal (period 2003–2018). (g ingredient needed / 100g finished product)—cut-off 0.15%.

Ingredients	2003 Recipe	2010 Recipe	2018 Recipe
Refined wheat flour	35.6	17.7	23.0
Whole wheat flour		34.6	41.3
Corn semolina	13.5		
Rice flour		6.29	
Sugar	28	26.1	21.3
Glucose syrup	8.80	6.85	11.8
Cocoa powder/mass	2.25	1.99	2.54
Palm oil	7.23	5.18	
Sunflower oil			5.58
Caramel paste	7.97	8.52	
Skim milk powder	2.10		
Sweetened condensed milk			3.23
Dextrose	1.18	1.02	
Calcium carbonate	1.38	0.68	1.18
Salt	0.59	0.51	0.49

Table S4. Sourcing of materials.

Material	Source	Mode of Transportation	Distance (km)
Refined wheat flour	France	Truck, >32 ton	250
Whole wheat flour	France	Truck, >32 ton	250
Rice flour	Europe	Truck, >32 ton	1000
Corn semolina	Europe	Truck, >32 ton	1000
Wheat starch	Europe	Truck, >32 ton	1000
Sugar	France	Truck, >32 ton	250
Dextrose	Europe	Truck, >32 ton	1000
Glucose syrup	Europe	Truck, >32 ton	1000
Sweetened condensed milk	Europe	Truck, >32 ton	1000
Caramel paste	Europe	Truck, >32 ton	1000
Cocoa powder	East Africa	Truck, >32 ton	1000
		Transoceanic ship	10 000
Palm oil & palm kernel oil	South East Asia	Truck, >32 ton	1000
		Transoceanic ship	10 000
Sunflower oil	France	Truck, >32 ton	250
Calcium carbonate	Europe	Truck, >32 ton	
Salt	France	Truck, >32 ton	250
Soya lecithin	United States	Truck, >32 ton	1000
		Transoceanic ship	6 000
Sunflower lecithin	France	Truck, >32 ton	250
Malt extract	Europe	Truck, >32 ton	1000
Skimmed milk powder	Europe	Truck, >32 ton	1000
HDPE bag	Europe	Truck, >32 ton	1080
Folding board box (FBB)	Europe	Truck, >32 ton	1080
	France	Truck, >32 ton	384
Corrugated board case	France	Truck, > 32ton	250
LLDPE Stretch film	Europe	Truck, > 32ton	1080

Table S5. Packaging system composition (g material/breakfast cereal box).

Packaging Material	Chocapic (430 g)			Lion (480 g)			Nesquik (450 g)		
	2003	2010	2018	2003	2010	2018	2003	2010	2018
HDPE bag	9.80	8.70	8.20	9.40	10.10	9.60	10.80	9.50	9.00
Folding board box (FBB)	52.40	45.60	44.70	55.40	48.20	47.40	59.60	54.20	53.30
Corrugated board case	23.30	21.20	19.30	20.90	19.00	16.80	25.70	23.30	21.20
LLDPE Stretch film	0.84	0.76	0.69	0.69	0.63	0.57	0.91	0.82	0.75
Boxes per pallet	576			704			432		

Table S6. Manufacturing processes data (per kg of product).

Inputs	Consumption 2003		Consumption 2010		Consumption 2018	
	Factory 1	Factory 2	Factory 1	Factory 2	Factory 1	Factory 2
Electricity (France) (MJ)	4.86	4.72	4.58	4.16	2.17	1.52
Heat (natural gas) (MJ)	3.18	5.76	4.32	2.96	4.42	2.89
Water (l)	12.30	9.60	3.57	3.05	5.20	3.16

Table S7. End of life options for packaging materials in France.

Material	Year	SL	I	IER	R	C
Municipal solid waste	2003	38.0%	2.2%	31.8%	15.0%	13.0%
	2010	29.1%	1.2%	33.6%	20.0%	16.0%
	2018	20.9%	0.3%	34.8%	25.1%	18.9%
Plastic packaging	2003	43.9%	2.5%	36.7%	17.0%	-
	2010	36.0%	1.5%	41.5%	21.0%	-
	2018	27.5%	0.3%	45.7%	26.5%	-
Cardboard	2003	30.6%	1.7%	25.6%	42.0%	-
	2010	20.0%	0.8%	23.1%	56.0%	-
	2018	12.0%	0.1%	19.9%	68.0%	-

CC: Composting; I: Incineration without energy recovery; IER: Incineration with energy recovery; R: Recycling; SL: Sanitary landfill.

Table S8. Nutritional content of all breakfast cereal recipes.

Product	Cereal 1			Cereal 2			Cereal 3		
Year	2003	2010	2018	2003	2010	2018	2003	2010	2018
Nutritional Labels / 100 g									
Energy (kcal)	392	393	385	394	379	369	422	407	408
Protein (g)	7.7	8.4	8.5	5	7.3	8.4	6.4	7.4	8.1
Carbohydrate (g)	79.9	76	73.4	83.6	79.1	75.8	77.5	76.7	72.8
Sugars (g)	42	37	24.9	38	35	24.9	39.1	35	25
Fat (g)	4.6	4.8	4.6	4.4	3.8	1.7	9.6	7.8	7.9
Saturated fat (g)	2.8	2	1.3	3	1.6	0.6	4.8	3.2	1.4
Fibre (g)	3.5	5.9	8	2.4	5.1	8.7	1.7	4.3	6.7
Sodium (g)	0.2	0.1		0.3	0.2		0.3	0.2	
Salt (g)			0.33			0.44			0.49
Nutri-Score (Letter/Score)	D/14	C/9	C/6	D/15	C/9	B/0	E/19	D/13	C/8

Table S9. Relative contribution of life cycle stages to overall impacts for Cereal 1.

Year	Indicator	Ingredients	Ingredients Sourcing	Packaging	Manufacture	Distribution	Storage and Retail	Packaging End of Life
2003	ARD	29%	3%	22%	38%	4%	5%	-1%
2010		34%	3%	24%	28%	5%	7%	-2%
2018		40%	4%	24%	22%	5%	7%	-2%
2003	CC	68%	1%	8%	16%	2%	2%	2%
2010		74%	1%	8%	10%	2%	3%	1%
2018		75%	1%	8%	9%	2%	3%	1%
2003	FWCS	93%	0%	5%	3%	0%	1%	-2%
2010		95%	0%	4%	2%	0%	1%	-2%
2018		98%	0%	2%	1%	0%	0%	-1%
2003	IEEQ	85%	1%	7%	4%	1%	2%	0%
2010		87%	1%	6%	3%	1%	2%	0%
2018		88%	1%	6%	2%	1%	2%	0%
2003	LUIB	93%	0%	9%	0%	0%	0%	-3%
2010		95%	0%	8%	0%	0%	0%	-3%
2018		96%	0%	7%	0%	0%	0%	-3%

ARD: Abiotic resource depletion; CC: Climate change; FWCS: Freshwater consumption scarcity; IEEQ: Impact on ecosphere/ecosystems quality; LUIB: Land use impact on biodiversity.

Table S10. Relative contribution of life cycle stages to overall impacts for Cereal 2.

Year	Indicator	Ingredients	Ingredients Sourcing	Packaging	Manufacture	Distribution	Storage and Retail	Packaging End of Life
2003	ARD	30%	5%	25%	31%	5%	6%	-1%
2010		31%	4%	22%	34%	5%	6%	-1%
2018		34%	3%	22%	32%	5%	6%	-2%
2003	CC	66%	2%	10%	14%	2%	3%	2%
2010		67%	2%	9%	16%	2%	3%	2%
2018		68%	1%	9%	15%	2%	3%	1%
2003	FWCS	95%	0%	3%	3%	0%	0%	-1%
2010		97%	0%	2%	1%	0%	0%	-1%
2018		98%	0%	2%	1%	0%	0%	-2%
2003	IEEQ	84%	1%	7%	4%	1%	2%	0%
2010		86%	1%	6%	4%	1%	2%	0%
2018		87%	1%	7%	3%	1%	2%	0%
2003	LUIB	87%	0%	18%	1%	0%	0%	-6%
2010		93%	0%	11%	0%	0%	0%	-5%
2018		95%	0%	9%	0%	0%	0%	-4%

ARD: Abiotic resource depletion; CC: Climate change; FWCS: Freshwater consumption scarcity; IEEQ: Impact on ecosphere/ecosystems quality; LUIB: Land use impact on biodiversity.

Table S11. Relative contribution of life cycle stages to overall impacts for Cereal 3.

Year	Indicator	Ingredients	Ingredients Sourcing	Packaging	Manufacture	Distribution	Storage and Retail	Packaging End of Life
2003	ARD	31%	4%	19%	38%	4%	5%	-1%
2010		32%	4%	24%	30%	6%	6%	-2%
2018		45%	3%	21%	22%	5%	6%	-2%
2003	CC	60%	2%	9%	21%	2%	3%	2%
2010		60%	2%	11%	17%	3%	4%	2%
2018		70%	2%	10%	11%	3%	3%	1%
2003	FWCS	96%	0%	3%	2%	0%	0%	-1%
2010		95%	0%	4%	2%	0%	1%	-3%
2018		99%	0%	1%	0%	0%	0%	-1%
2003	IEEQ	86%	1%	6%	4%	1%	2%	0%
2010		86%	1%	6%	3%	1%	2%	0%
2018		91%	1%	5%	2%	1%	1%	0%
2003	LUIB	93%	0%	10%	0%	0%	0%	-3%
2010		94%	0%	9%	0%	0%	0%	-4%
2018		98%	0%	4%	0%	0%	0%	-2%

ARD: Abiotic resource depletion; CC: Climate change; FWCS: Freshwater consumption scarcity; IEEQ: Impact on ecosphere/ecosystems quality; LUIB: Land use impact on biodiversity.
